# *cellXpress*: a fast and user-friendly software platform for profiling cellular phenotypes

**DOI:** 10.1186/1471-2105-14-S16-S4

**Published:** 2013-10-22

**Authors:** Danai Laksameethanasan, Rui Zhen Tan, Geraldine Wei-Ling Toh, Lit-Hsin Loo

**Affiliations:** 1 Bioinformatics Institute, Agency for Science, Technology and Research, 30 Biopolis Street, #07-01 Matrix, Singapore 138671, Singapore; 2 School of Computer Engineering, Nanyang Technological University, Nanyang Avenue, Singapore 639798, Singapore; 3 Department of Pharmacology, Yong Loo Lin School of Medicine, National University of Singapore, 10 Medical Drive, Singapore 117597, Singapore

## Abstract

**Background:**

High-throughput, image-based screens of cellular responses to genetic or chemical perturbations generate huge numbers of cell images. Automated analysis is required to quantify and compare the effects of these perturbations. However, few of the current freely-available bioimage analysis software tools are optimized for efficient handling of these images. Even fewer of them are designed to transform the phenotypic features measured from these images into discriminative profiles that can reveal biologically meaningful associations among the tested perturbations.

**Results:**

We present a fast and user-friendly software platform called "*cellXpress*" to segment cells, measure quantitative features of cellular phenotypes, construct discriminative profiles, and visualize the resulting cell masks and feature values. We have also developed a suite of library functions to load the extracted features for further customizable analysis and visualization under the R computing environment. We systematically compared the processing speed, cell segmentation accuracy, and phenotypic-profile clustering performance of *cellXpress *to other existing bioimage analysis software packages or algorithms. We found that *cellXpress *outperforms these existing tools on three different bioimage datasets. We estimate that *cellXpress *could finish processing a genome-wide gene knockdown image dataset in less than a day on a modern personal desktop computer.

**Conclusions:**

The *cellXpress *platform is designed to make fast and efficient high-throughput phenotypic profiling more accessible to the wider biological research community. The *cellXpress *installation packages for 64-bit Windows and Linux, user manual, installation guide, and datasets used in this analysis can be downloaded freely from http://www.cellXpress.org.

## Introduction

High-throughput, image-based phenotypic profiling enables multi-parameter measurements of cellular responses to large-scale genetic or chemical perturbations. These measurements are useful for unraveling complex changes in cellular morphology and protein subcellular localization [1], and have been used to study drug responses [2], cell division [3], cytoskeleton remodelling [4], and endocytosis [5]. Several free software tools are currently available for analyzing microscopy images. They include CellProfiler [6], ImageJ [7], BioImageXD [8], Icy [9], OMERO [10], and EBImage [11].

**Figure 1:**
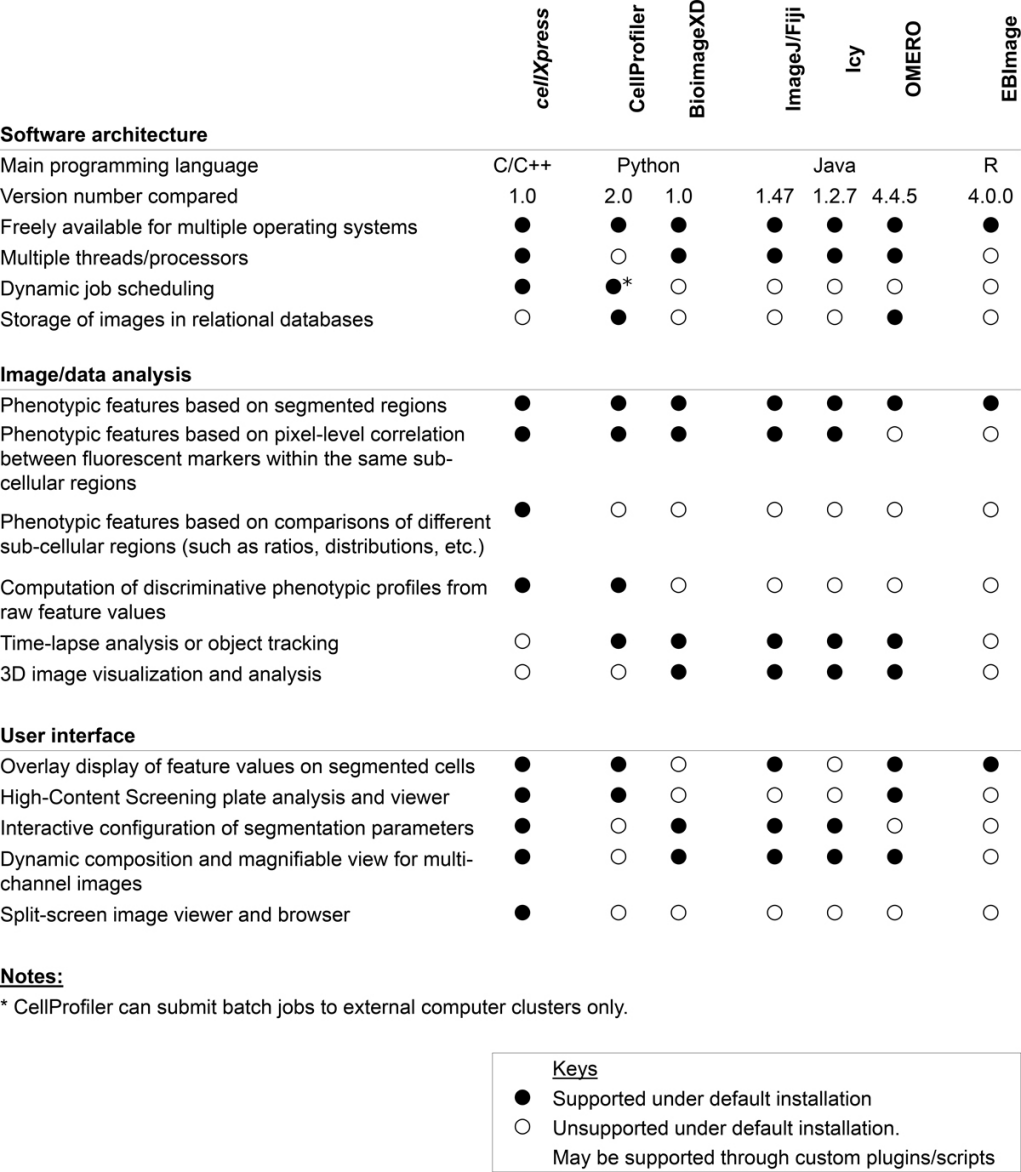
**Major functional differences between *cellXpress* and other existing bioimage analysis software platforms**.

However, most of these existing software tools are designed for general 2D, 3D or time-lapse image analyses, such as de-convolution, segmentation, registration, and motion tracking (Figure 1). Few of them are specifically designed for high-throughput cellular phenotype profiling that generates huge numbers of microscopy images (on the order of 10^4^-10^5^) and poses several new and different challenges to the analysis pipeline and user-interface design. First, individual cells need to be identified and quantified from these images within a reasonable time (usually less than a day for an entire dataset). However, most of the existing free bioimage analysis software tools are developed in high-level programming languages, such as Python or Java, which are slower than C/C++ in executing computational algorithms [12,13]. Some of the current tools alleviate this problem through external computer clusters [6], which are usually expensive and difficult to manage. Second, diverse types and often large numbers of phenotypic features are required to distinguish the effects of different perturbations [2]. However, most existing tools can extract limited types of phenotypic features. For example, features comparing different sub-cellular regions, such as nuclear versus cytoplasmic intensity, are not available in most existing tools without custom scripting or programming (Figure 1). Third, computational algorithms are required to transform the extracted features into discriminative profiles that can reveal biologically meaningful associations among the tested perturbations [14]. Very few of the existing tools can currently perform this function (Figure 1). Last, intuitive user interfaces are required for configuring algorithms and visualizing results, such as displaying the computed segmentation boundaries or feature values on top of cell images. Therefore, there is a need for a new free and user-friendly software tool that can address these needs of high-throughput phenotypic profiling.

## Implementation

### Overall software design and architecture

**Figure 2:**
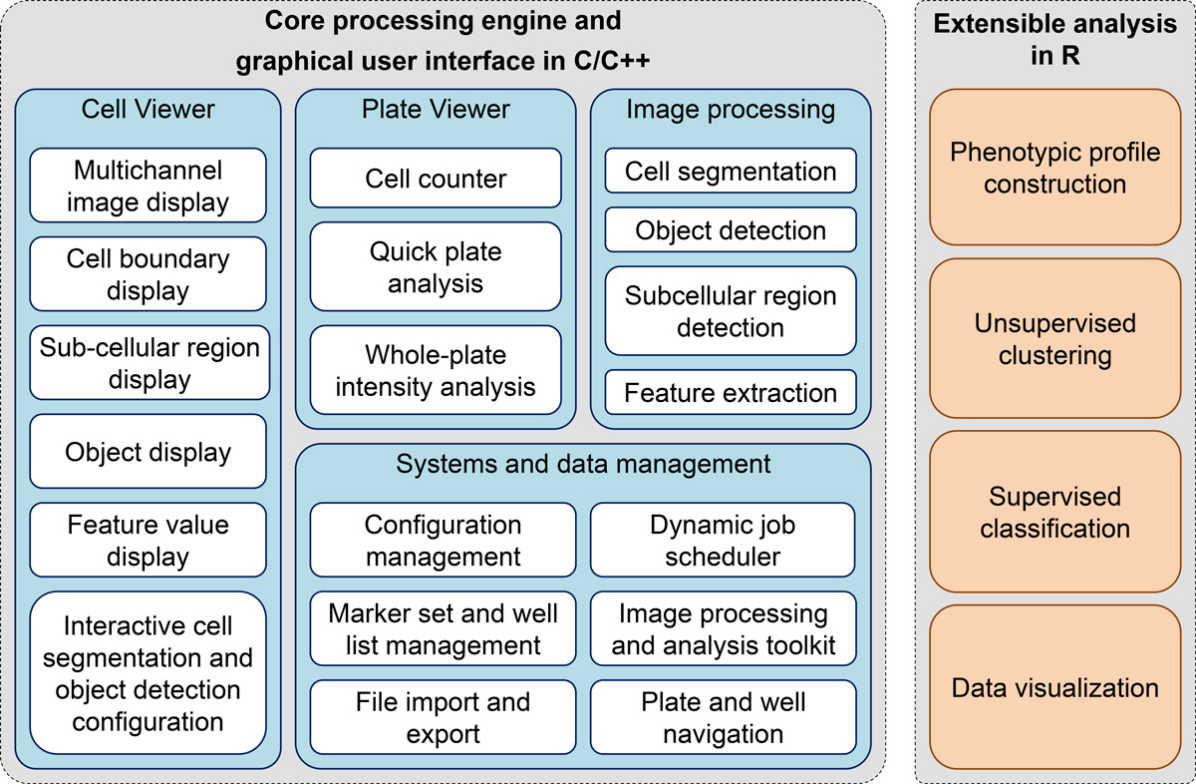
**The *cellXpress* cellular phenotype profiling software platform has two main frameworks**.

**Figure 3:**
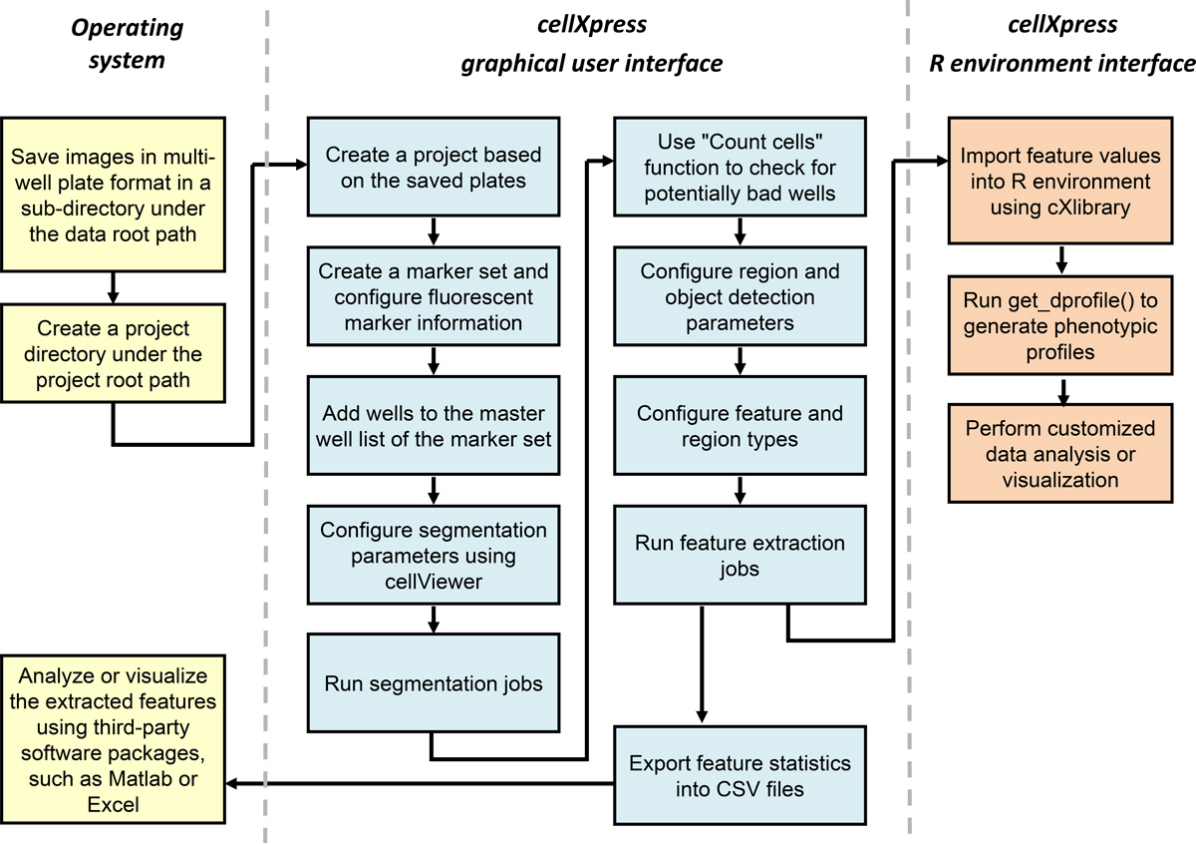
**Typical data analysis workflow for *cellXpress***.

Here, we present a new cell image analysis software platform called "*cellXpress*" for high-throughput phenotypic profiling. The platform consists of two main frameworks (Figure 2). The core framework is used for cell segmentation, feature extraction, image management and browsing, and quick data analysis (Figure 3). The second extensible framework is used for custom data analysis, including phenotypic profile construction and visualization (Figure 3). The *cellXpress *platform can read standard 8-bit or 16-bit TIFF or PNG images produced by most microscopy imaging systems. All the segmentation and feature extraction results computed by the core processing engine are saved in HDF5 binary files [15]. They can also be exported as standard 16-bit TIFF/PNG images or CSV files, which can be opened by third-party image viewers, spreadsheets or data analysis software packages for further processing.

The *cellXpress *platform has the following advantages over existing bioimage analysis software packages. First, to achieve higher processing speeds, we have developed the core processing engine of *cellXpress *completely in C/C++ based on a highly-optimized computer vision library, OpenCV [16], and efficient single-instruction-multiple-data (SIMD) instruction sets [17]. To fully utilize all the processing units in modern multi-core processors, we have also developed a dynamic job scheduler based on the OpenMP interface [18]. The scheduler manages a job queue for cell segmentation and feature extraction jobs, and automatically assigns pending jobs to free processors or cores. Thus, all the available processors will be fully ultilized by *cellXpress*.

Second, to extract diverse types of features, we have developed a new subcellular region detection algorithm that can automatically identify eight different subcellular regions based on the nuclear and cell masks from each segmented cell. These sub-cellular regions include whole-cell, nucleus, nuclear boundary, inner nucleus, peri-nucleus, cytoplasm, cytoplasmic boundary, and inner cytoplasm. The *cellXpress *platform automatically computes four different feature types, namely morphology, intensity, region-level intensity ratio and pixel-level intensity correlation, for each of the identified regions (see **Feature Extraction**). These diverse types of features will allow the quantification of complex protein subcellular localization patterns at the single-cell level.

Third, to perform profile construction or other custom data analyses, we have developed a suite of library functions called "cXlibrary" under the R computing environment [19]. Users can import *cellXpress *data saved in HDF5 files into a R session and construct phenotypic profiles. The resulting data or profiles can then be used for supervised classification, unsupervised clustering, or other types of analysis. We also implemented phenotypic profiling algorithms to condense large numbers of raw features extracted in typical high-throughput studies into more concise and discriminative profiles, such as the support-vector-machine (SVM)-based "drug profiles" (d-profiles) [2,14].

**Figure 4:**
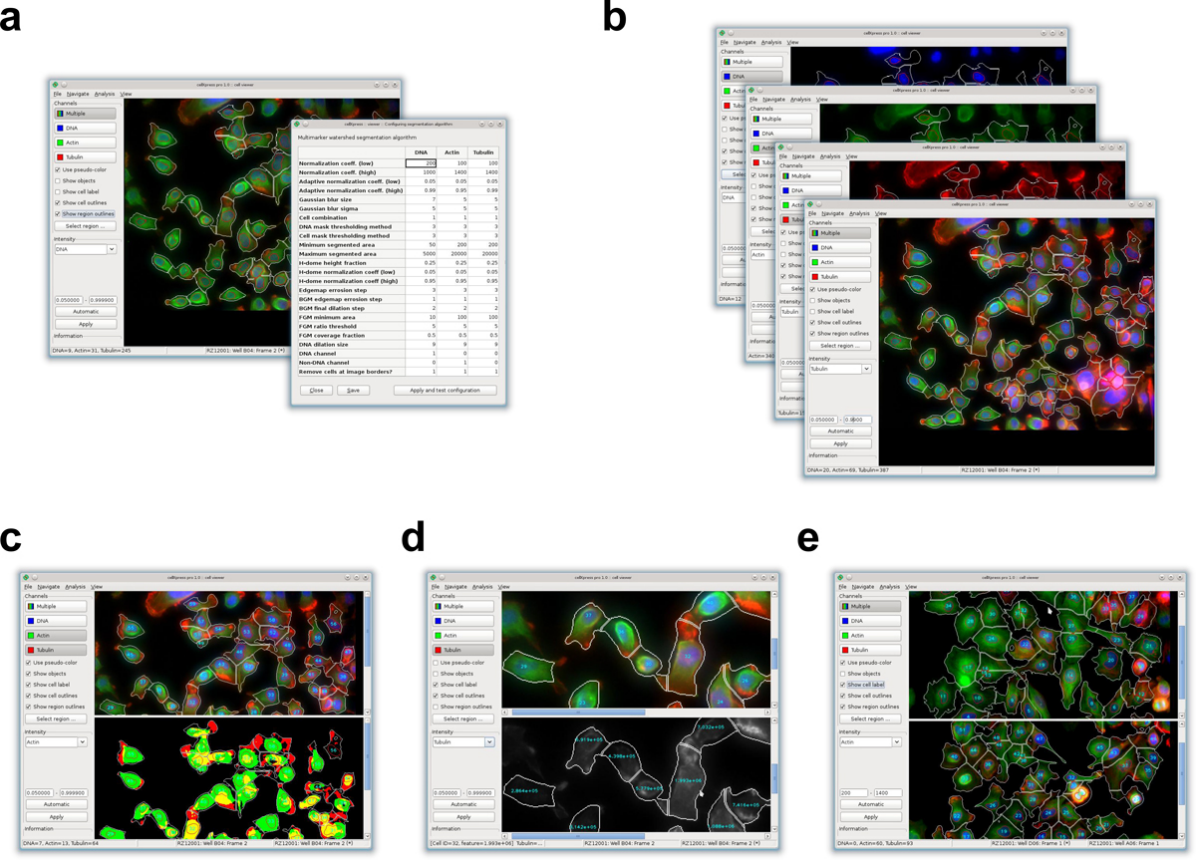
**Graphical User Interface of *cellXpress***. A common user interface is used to (a) interactively adjust segmentation parameters and display the resulting cellular outlines, (b) dynamically compose and display multi-channel fluorescence images, (c) visualize and compare results of object detection, (d) overlay display of extracted feature values on top of original images, and (e) browse and compare images from different wells.

**Figure 5:**
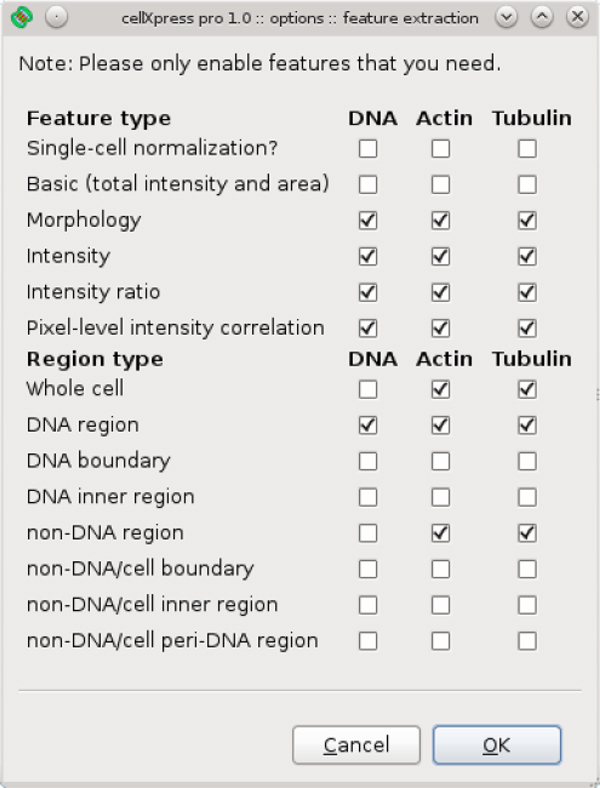
**Point-and-click interface to configure feature and region types**.

Finally, to make our software user-friendly, we have developed intuitive and interactive graphical user interfaces for configuring and controlling the image processing engine, and visualizing cell segmentation and feature extraction results (Figure 4). We have also designed a point-and-click interface to allow flexible configuration of feature extraction based on different combinations of feature types, sub-cellular regions, and fluorescent markers (Figure 5). Our graphical user interfaces are based on the cross-platform wxWidgets library [20], which provides a consistent look and feel across the Windows and Linux operating systems. Together, all of these components of *cellXpress *make it a fast and user-friendly software platform for high-throughput image-based phenotypic profiling.

### Seeded-watershed-based cell segmentation algorithm

We have implemented and optimized a seeded-watershed-based cell segmentation algorithm [2] for the *cellXpress *platform. The segmentation algorithm is general and was previously used to identify individual mouse fat cells, human cancer cells, and neutrophil-like cells from fluorescence microscopy images [2,21,22]. In brief, the algorithm consists of two major steps. The first step is to identify nuclear regions from the image background using a combination of h-dome operator [23], Laplacian-of-Gaussian edge detector, and Otsu's thresholding algorithm [24]. Then, a watershed algorithm [25] is used to break apart connected nuclear regions. In the second step, a composite cell image obtained from the linear combination of the images of all fluorescence channels is used to identify cellular regions based on Otsu's thresholding algorithm. Finally, a seeded watershed algorithm that uses the nuclear regions as seeds is used to break apart connected cellular regions. In the future, we plan to include additional segmentation algorithms in the *cellXpress *platform.

### Feature extraction

The *cellXpress *platform has a flexible feature extraction module that can be used to measure cellular features based on different combinations of feature types, fluorescent markers, and subcellular regions. We have designed a user-friendly graphical interface to configure this feature extraction module (Figure 5) and automatically expand feature selections based on the chosen regions. For example, region-level intensity ratios will be computed for all possible pairs of the selected sub-cellular regions. Information about the markers and regions used in a feature is encoded in its name, which follows the following format: XXXX:YYYY:ZZZZ, where XXXX is the feature type, YYYY is the fluorescent markers used, and ZZZZ is the subcellular regions used. For example, "fraction_total_intensity:ERK:dna_region-cell_region" measures the fraction of total ERK intensity level in the nuclear region to the entire cellular region, and "total_intensity_ratio:Tubulin-ERK:nondna_region-dna_region" measures the ratio of total tubulin intensity level in the cytoplasmic region over total ERK intensity level in the nuclear region. The names of the extracted features are included in all *cellXpress *HDF5 data files or CSV export files. Users can load specific subsets of extracted features into the R environment by using the load_wells() function in the cXlibrary.

### Phenotypic profiling

To transform extracted features into discriminative profiles, we implemented a support-vector-machine-(SVM)-based phenotypic profiling algorithm called "drug profiling" (d-profiling) [2] in the *cellXpress *platform. Given two sets of feature values for cells under treated and control conditions, respectively, the algorithm trains a linear SVM to obtain a hyperplane that optimally separates these two set of values in high-dimensional feature space. Then, the unit vector normal to the hyperplane is used as a profile to represent changes in the phenotypes of the treated cells with respect to the control cells [2]. Our implementation is based on the LIBLINEAR library [26].

## Evaluation methods

### Comparisons with existing tools

To evaluate the performance of *cellXpress*, we considered several other alternative free biological image analysis software platforms (Figure 1). The functions of many of these platforms may be extended through third-party plugins or custom scripting/programming. However, most biological scientists will have limited resources or expertise in developing such custom plugins or programs. Therefore, we only considered built-in functions or plugins that are bundled with default installation packages. We chose to compare the performance of *cellXpress *(version pro 1.0) [27] to the Broad Institute's CellProfiler (version 2.0) [28] because they have the most similar functions (Figure 1). We also included NIH's ImageJ (version 1.47) [29] with plugins from the Fiji package [30] because it is a standard image analysis tool and widely used by biological scientists. We focused on evaluating the processing speed, cell segmentation accuracy, and profile clustering performance of these software packages.

### Applications to Kc167, HT29, and HeLa datasets

To evaluate cell segmentation performance of *cellXpress*, we used two standard image benchmark datasets, namely Kc167 and HT29, which represent different cell types and numbers of image frames [31,32]. The first dataset was collected from a *Drosophila melanogaster *cell line, Kc167. We used the dataset's DNA marker for detecting nuclear regions, and actin marker for detecting cellular regions. This dataset has three image frames (an image frame refers to an imaging position in a well), but we only used one of them for testing cell segmentation speed to mimic the situation when computation cannot be parallelized at the image-frame level. Each image has a resolution of 1000 × 1006 pixels, and there are ~200 cells per frame. The second dataset [33] was collected from a human colon cancer cell line, HT29. We only used the dataset's DNA marker for detecting nuclear regions, and actin marker for detecting cellular regions. The dataset was generated in a shRNA screen for finding mitotic gene regulators [33]. It has 56 image frames, and was used to test cell segmentation when computation may be parallelized at the frame-level. Each image has a resolution of 512 × 512 pixels, and there are ~100 cells per frame. We followed the procedures recommended on the CellProfiler website [31], and used the original images and the provided pipeline without any further image pre-processing.

To evaluate the phenotypic-profiling performance of *cellXpress*, we used an image dataset from a previous high-throughput siRNA screen [34,35] on HeLa cells stained for DNA, tubulin, and actin markers. The dataset was generated by transfecting HeLa cells with a genome-wide siRNA library for 48 hours, and used to predict functions of genes based on their knockdown phenotypes. There are four 670 × 510 pixel image frames per gene knockdown, each of which has around 50 cells. siRNAs for a non-human gene, renilla luciferase (Rluc), were used as negative controls. We selected 32 genes, which can be categorized into four groups representing structural components of actins or microtubules, or the synthesis machineries for RNAs or proteins (Additional file 1). The RNA and protein synthesis genes were selected from genes encoding the subunits of RNA polymerase II and ribosome, respectively. Microtubule structural components were selected from the α-tubulin, β-tubulin and γ-tubulin families. For structural components of actins, we included three actin isoforms (alpha, beta and gamma) and genes from the spectrin family, which are actin-crosslinking proteins that link the plasma membrane to the actin cytoskeleton [36].

### Evaluation criteria for segmentation accuracy

We used two different segmentation accuracy criteria: the boundary and Rand error indices [37]. The boundary error index ($E_{\text{boundary}}$) measures the averaged distance between the boundaries of cellular masks obtained from manual and automated segmentation, respectively. Smaller boundary error index values mean higher automated segmentation accuracy. We define the boundary error index between two sets of boundary pixels ( B and $B^{\prime }$) from a manual segmentation mask ( M) and an automated segmentation mask ($M^{\prime }$), respectively, to be:

$$E_{\text{boundary}}\left(M,M^{\prime }\right)=\frac{1}{\left|B\right|}\underset{b\in B}{\sum }\underset{b^{\prime }\in B^{\prime }}{\text{min}}\left\{∥b-b^{\prime }{∥}^{2}\right\},$$

where  b and $b^{\prime }$ are individual pixels within sets  B and $B^{\prime }$, respectively;  is the cardinality operator; and  is the Euclidean norm.

We also used the Rand error index [37], which measures the frequency with which the two segmentation masks disagree over whether a pair of pixels belongs to same or different segmented cellular regions. Let the set of labelled regions in a manual segmentation mask be $L=\left\{R_{i}\right\}$ and the set of labelled regions in an automated segmentation mask be $L^{\prime }=\left\{R_{j}^{\prime }\right\}$, where $R_{i}$ and $R_{j}^{\prime }$ are the *i*-th and *j*-th connected pixels within the respective masks. Furthermore, we denote  c as the number of pixel pairs in  M that belongs to the same sets in  L and the same sets in $L^{\prime }$, and  d as the number of pixel pairs in  M that belongs to different sets in  L and different sets in $L^{\prime }$. Then, the Rand error index is:

$$E_{\text{Rand}}\left(M,M^{\prime }\right)=1-\frac{c+d}{\left(\begin{array}{c} N\\ 2 \end{array}\right)}.$$

where *N *is the total number of pixels in the segmentation mask *M*.

### Generation of phenotypic profiles for HeLa dataset

To construct phenotypic profiles for HeLa cells, we first segmented the dataset using *cellXpress*. Actin and tubulin were used as cell markers and DNA as a nuclear marker for the watershed algorithm. Then, we measured the morphology, intensity, intensity ratio, and pixel-level intensity correlation features for actin and tubulin in the whole cell, nuclear and non-nuclear regions; and for DNA in the nuclear region only. In total, we measured 290 features for every cell (Additional file 2). Then, we constructed three different types of phenotypic profiles for the dataset. The first type of profiles is based on the arithmetic mean of each feature across all cells that have been treated with a specific siRNA. The second type of profiles is based on principal component analysis (PCA) [38]. We kept the number of principal components needed to explain 95% of the variation in our data, and used the scores vector as the phenotypic profiles. The last type of profiles is the SVM-based "d-profiles" [2] (see **Implementation Section**).

### Evaluation criteria for phenotypic profiling

To evaluate the performance of these three phenotypic profiling methods, we measured the intra-group and inter-group dissimilarities for the four groups of siRNAs (Additional file 1). Other criteria based on centroids or medoids of the groups are not suitable for this dataset, because most of the profiles have highly-asymmetrical and non-Gaussian-like distributions. We computed the cosine dissimilarity between two profiles $g_{r}$ and $g_{s}$ as:

$$d\left(g_{r},g_{s}\right)=1-\frac{g_{r}g_{s}^{T}}{\sqrt{\left(g_{r}^{T}g_{r}\right)\left(g_{s}^{T}g_{s}\right)}},$$

where $g^{T}$ is the vector transpose of  g. To determine the average 'compactness' of profiles within a group, we computed the average maximum intra-group dissimilarity score as:

$$D_{\text{intra}}=\frac{1}{N}\overset{N}{\underset{j=1}{\sum }}\underset{g_{r},g_{s}\in G_{j}}{\text{max}}\left\{d\left(g_{r},g_{s}\right)\right\},$$

where $G_{j}$ is the set of all profiles in the *j-*th group, and  N is the total number of groups.

To determine the average inter-group profile dissimilarity, we first sorted all pair-wise dissimilarities between profiles from two different groups, $G_{j}$ and $G_{k}$ from the lowest to the highest, where $d_{1}<d_{2}<d_{3}<d_{4}<\ldots$, and $d_{i}=d\left(g_{r},g_{s}\right)$ for all $g_{r}\in G_{j}$ and $g_{s}\in G_{k}$. For a *n*-nearest neighbours analysis, we denote the set of *n *lowest distances between two groups, $G_{j}$ and $G_{k}$, as $W_{jk}\left(n\right)=\left\{d_{1},d_{2},d_{3},\ldots ,d_{n}\right\}$. Then, the inter-group profile dissimilarity for the *n*-nearest neighbours is:

$$D_{\text{inter}}=\frac{2}{N\left(N-1\right)}\overset{N}{\underset{j=1}{\sum }}\underset{k\neq j}{\sum }E\left(W_{jk}\left(n\right)\right),$$

where *E*() is the mean operator. This evaluation is repeated for different values of *n*.

#### Computer software and hardware platforms

The evaluations were performed on a desktop computer with a Intel Core i7 3.07 GHz processor, 8 GB of memory, 64-bit Windows 7 operating system, and Java version 7 Update 9 (build 1.7.0_09-b05). All image and data files were stored in a local harddrive. For the evaluation of processing speed and segmentation accuracy, we implemented a script in Matlab version R2007b (Mathworks, USA) to compute and compare both the boundary and Rand error indices. For the evaluation of phenotypic profiling, we generated multidimensional scaling (MDS) plots for all the constructed profiles using the MASS [40] and the rgl libraries [41] under the R computing environment (version 2.14.2).

## Results and discussion

### Processing speed and segmentation accuracy

A fast and accurate bioimage analysis software platform is required to process the huge amount of microscopy images generated from high-throughput phenotypic profiling experiments. We compared the processing speed and accuracy of *cellXpress*, CellProfiler [6], and ImageJ/Fiji [42] in segmenting the Kc167, HT29, and HeLa datasets. These three software packages implement variants of similar seeded watershed segmentation algorithms [25]. For CellProfiler [43], we used the segmentation pipeline and optimized parameters included in the Kc167 dataset zip file [31]. We disabled the image cropping function, "show all windows on run" option, and feature-extraction steps in the original pipeline. The pipeline identified DNA regions and the cellular regions using Otsu's thresholding algorithm [43]. For Fiji [42], we implemented a macro script to perform watershed segmentation. The script identified cellular regions using Li's Minimum-Cross-Entropy [44] and Triangle-thresholding algorithms [45] implemented in Fiji for the Kc167 and HT29 datasets, respectively. We found that these two thresholding algorithms gave the best segmentation results for the respective datasets for Fiji.

**Figure 6:**
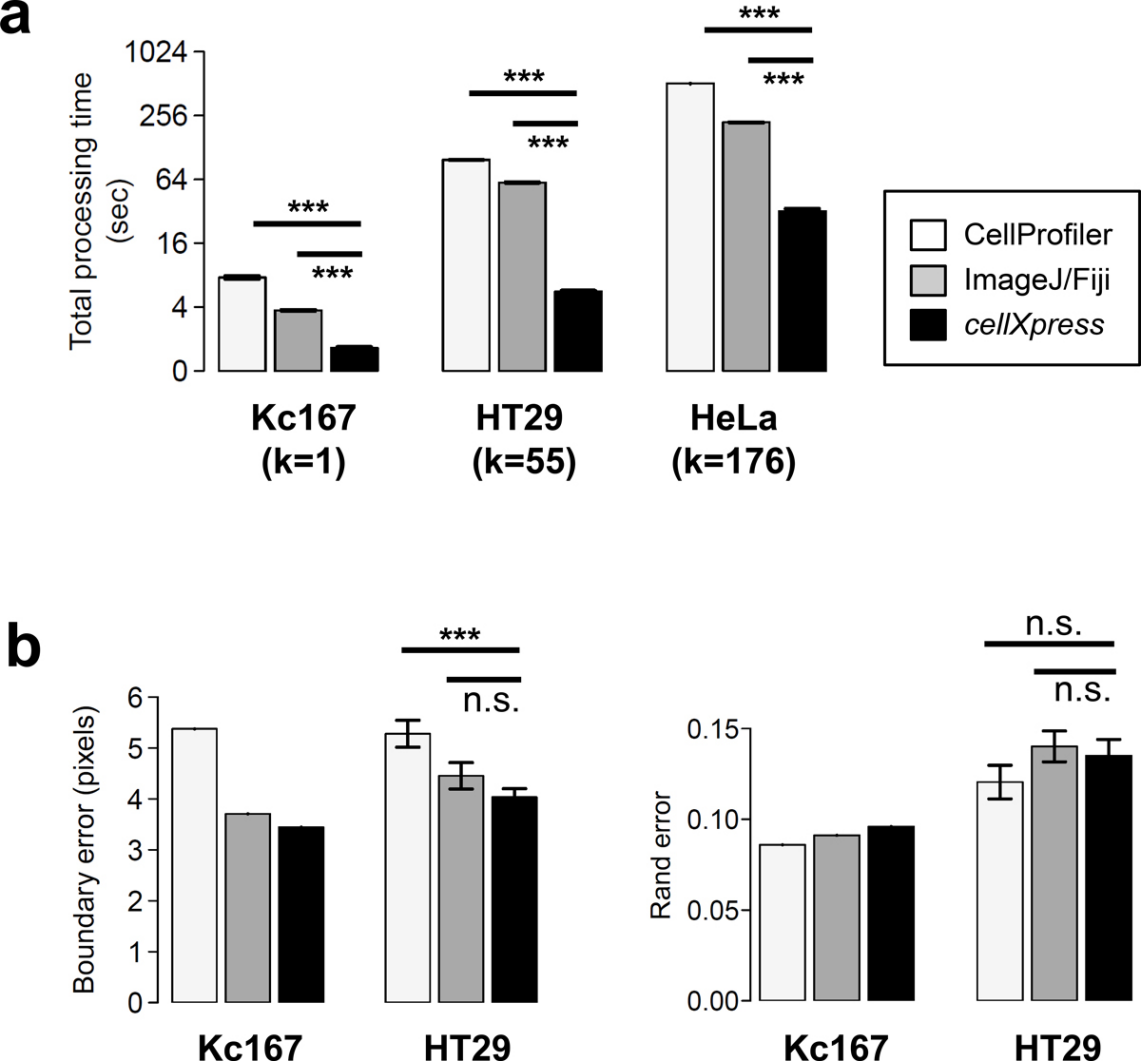
**Evaluation of processing speed and segmentation accuracy**. (a) Processing time of CellProfiler, ImageJ/Fiji, and *cellXpress* in segmenting Kc167, HT29 and HeLa image datasets. (b) Segmentation accuracy of CellProfiler, ImageJ/Fiji, and cellXpress. (k = total number of image frames, *** = P<0.001, n.s. = P>0.05; two-sided t-test).

To evaluate processing speed, we measured the processing time of the whole segmentation process, which includes image loading, processing, and saving. We repeated the measurement five times and computed the mean and standard error of the measurements (Additional file 3). To avoid memory caching, we re-started each software package after every measurement. We found that *cellXpress *was ~2.3-17.5 times faster than CellProfiler and Fiji (Figure 6a and Additional file 3) on the three tested datasets. For the Kc167 dataset with only one image frame, *cellXpress*, Fiji and CellProfiler needed 1.65, 3.7, and 7.6 seconds, respectively, to complete the segmentation jobs. For the HeLa dataset with 176 image frames, *cellXpress*, Fiji and CellProfiler took 32.1, 220.7, and 512.8 seconds respectively, to complete the segmentation jobs. This higher per image performance of *cellXpress *was partially due to the dynamics job scheduler in *cellXpress*. Based on our results, we estimate that *cellXpress *would only take ~5-9 hours to process a typical genome-wide gene knockdown image dataset (~20,000 genes × 9 frames/gene = ~180,000 frames) on a personal desktop similar to our test system, while other software tools could take ~2-6 days. The fast processing speed of *cellXpress *makes it more efficient for analyzing data generated from high-throughput experiments, such as gene-knockdown or small-molecule screens, on modern desktop computers without requiring expensive computer clusters.

To evaluate segmentation accuracy, we compared cell masks obtained automatically from the three software platforms to cell masks obtained from manual segmentation. For the Kc167 dataset, we manually segmented each individual cell based on the actin channel. For the HT29 dataset, we used the manual segmentation masks from the Broad Institute's website [32]. The image frame "10779.DIB" was excluded from analysis, as suggested from the website, because of insufficient image quality. We found that the *cellXpress *had slightly better or similar segmentation accuracies than Fiji and CellProfiler (Figure 6b). The boundary error of *cellXpress *was significantly lower than CellProfiler (P<0.001), but the Rand errors of the three tested tools were not significantly different from each other (P>0.05, both using two-sided t-tests). Therefore, the faster speed of *cellXpress *does not come at the cost of segmentation accuracy.

### Evaluation of phenotypic profiling

**Figure 7:**
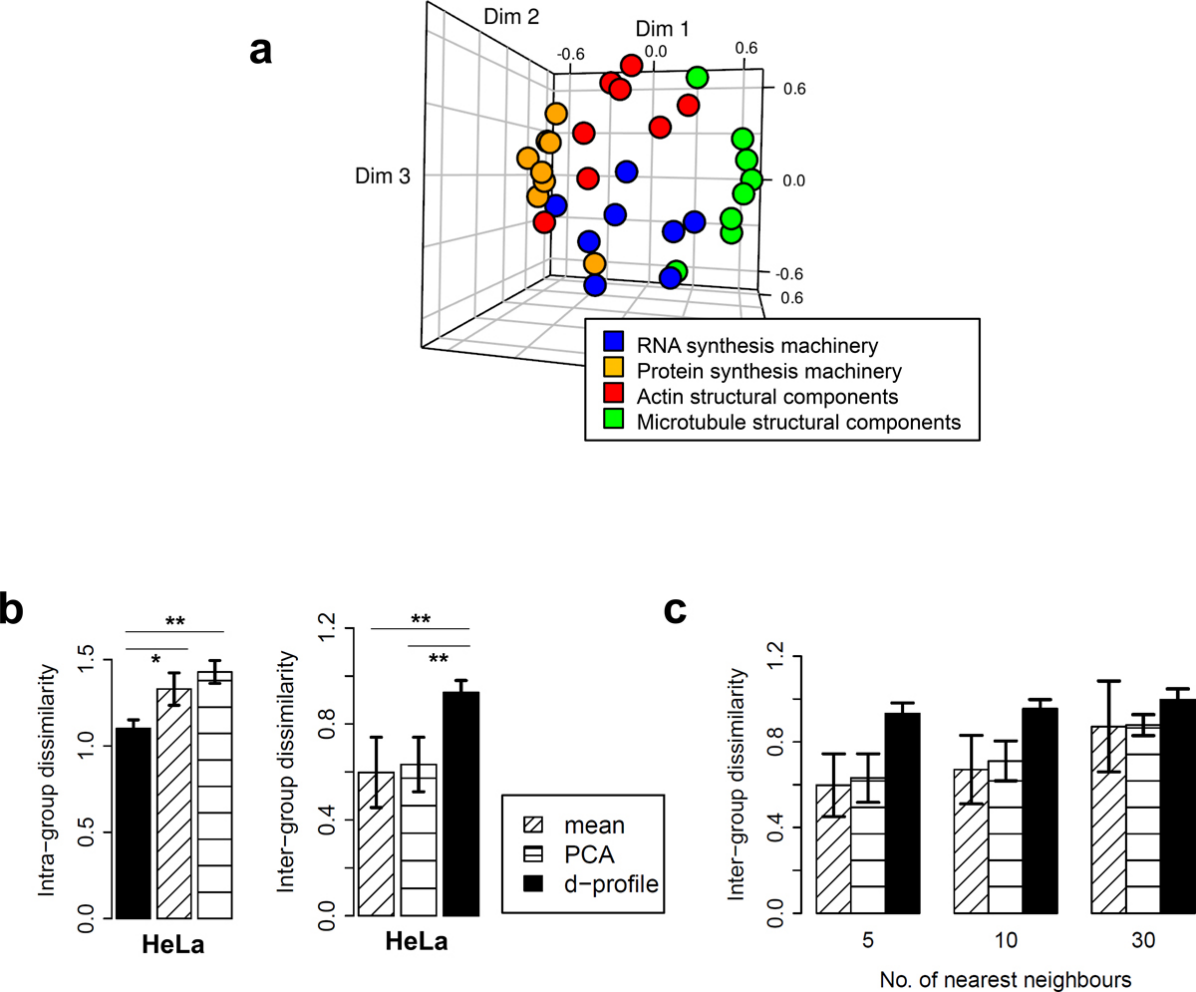
**Evaluation of phenotypic profiling**. (a) Multidimensional scaling plot based on the cosine dissimilarities among the d-profiles for the 32 siRNAs, which are color-coded according to their known biological functions. (b) Mean intra- and inter-group dissimilarities of phenotypic profiles constructed for 32 siRNAs in the HeLa dataset based on mean-, principal-component-analysis-(PCA)-based, and "d-profiles" (error bars = SEM, * = P<0.05, ** = P<0.01, *** = <0.001; two-sided paired t-test.). (c) Inter-group dissimilarities of mean-, PCA-based and d-profiles for different numbers of nearest neighbors. (Error bars = SEM).

To demonstrate the ability of *cellXpress *to identify functional relationships from large-scale gene knockdown studies, we considered an image dataset from a siRNA screen on HeLa cells stained for DNA, tubulin and actin [35]. We focused on four groups of genes that are part of the structural components of actins or microtubules, or the synthesis machineries for RNAs or proteins (Additional file 1); and constructed three types of phenotypic profiles, namely mean, PCA, and d-profiles, for the dataset (Figure 7a). We found that d-profiles separate these groups better, with smaller intra-group and larger inter-group average dissimilarity, than mean- or PCA-based profiles (Figure 7b). We tested *n *= 5, 10 and 30, and found that d-profiles had the highest average inter-group distance, irrespective of *n *(Figure 7c).

The better performance of d-profiles may be attributed to its ability to capture more informative features. Mean profiles are the arithmetic means of the extracted features across all cells, and PCA profiles are based on an orthogonal transformation of the features into a new set of linearly uncorrelated variables with descending variance (see **Evaluation Methods**). Both methods do not remove or penalize non-informative features that show high-variance but similar values in both siRNA-treated and control cells. However, d-profiles are based on SVM hyperplanes that optimally separate between treated and control cells, and thus will give lower weights to these non-informative features. Interestingly, we found that d-profiles could distinguish genes involved in the synthesis machineries of RNAs or proteins (Figure 7a), although the cells were only stained with markers for cytoskeleton components. This shows the potential of using morphological and intensity features of a small set of markers to distinguish genes with different biological functions.

## Conclusions

The *cellXpress *platform is specifically designed to make fast and efficient high-throughput phenotypic profiling more accessible to the wider scientific community. Other biological image analysis software platforms may be more appropriate for analyzing time-lapse or 3D microscopy images, or managing large image databases (Figure 1). The *cellXpress *platform is actively maintained and updated. Future planned improvements include graphics-processing-unit (GPU)-based acceleration, and gene or chemical annotation analysis. The *cellXpress *software package can be downloaded from http://www.cellXpress.org.

## Competing interests

The authors declare that they have no competing interests.

## Authors' contributions

LHL conceived the software architecture. LHL and DL designed and implemented the software packages. RZT and DL performed the performance comparisons and analyses. GWLT prepared the documentation and website. All authors participated in the writing of the manuscript, and approved the final manuscript.

## Supplementary Material

Additional file 1 **Plate layout for the genes in the RNA synthesis (blue), ribosomal (yellow), actin (red) and tubulin (green) groups**. Click here for file

Additional file 2 **Feature list for the HeLa siRNA dataset**. Click here for file

Additional file 3 Total processing time for cell segmentation (unit = second, k = frame number, CP = CellProfiler, cX = cellXpress) Click here for file

## Declarations

Publication of the work is supported by Biomedical Research Council of A*STAR (Agency for Science, Technology and Research), Singapore.

This article has been published as part of *BMC Bioinformatics *Volume 14 Supplement 16, 2013: Twelfth International Conference on Bioinformatics (InCoB2013): Bioinformatics. The full contents of the supplement are available online at http://www.biomedcentral.com/bmcbioinformatics/supplements/14/S16.
