# Supplementary material for: cellXpress: a fast and user-friendly software platform for profiling cellular phenotypes
Source: BMC Bioinformatics. 2013 Oct 22;14(Suppl 16):S4. doi: 10.1186/1471-2105-14-S16-S4 (PMC3853218; doi:10.1186/1471-2105-14-S16-S4)
Supplement: Additional file 1 — Plate layout for the genes in the RNA synthesis (blue), ribosomal (yellow), actin (red) and tubulin (green) groups. [file 1471-2105-14-S16-S4-S1.PDF]

Supplementary Table S1: Plate layout for the *genes* in the RNA synthesis (blue), ribosomal (yellow), actin (red) and tubulin (green) groups.

	1	2	3	4	5	6	7	8	9	10	11	12
**A**	A04 of Plate 11	B04 of Plate 11	POLR2A	POLR2B	POLR2G	TUBB6	TUBA1	TUBA2	TUBB1	TUBB4	TUBB4Q	TUBG1
**B**	A04 of Plate 25	B04 of Plate 25	POLR2F	POLR2H	POLR2I	POLR2J	POLR2K					
**C**	A04 of Plate 26	B04 of Plate 26	RPS10	RPS18	RPS20	RPS24	RPS12	RPS15A	RPS25	RPS26	SPTBN1	SPTBN2
**D**	A04 of Plate 12	B04 of Plate 12	ACTA1	ACTA2	ACTB	ACTG1	ACTG2					
**E**	A04 of Plate 34	B04 of Plate 34	SPTBN5									
**F**	A04 of Plate 10	B04 of Plate 10	TUBA3									
**G**												
**H**												
