# Supplementary material for: cellXpress: a fast and user-friendly software platform for profiling cellular phenotypes
Source: BMC Bioinformatics. 2013 Oct 22;14(Suppl 16):S4. doi: 10.1186/1471-2105-14-S16-S4 (PMC3853218; doi:10.1186/1471-2105-14-S16-S4)
Supplement: Additional file 2 — Feature list for the HeLa siRNA dataset. [file 1471-2105-14-S16-S4-S2.PDF]

Supplementary Table S2: Feature list for the HeLa siRNA dataset

area:mask:cell_region
filled_area:mask:cell_region
perimeter:mask:cell_region
major_axis_length:mask:cell_region
minor_axis_length:mask:cell_region
aspect_ratio:mask:cell_region
eccentricity:mask:cell_region
orientation:mask:cell_region
compactness:mask:cell_region
roundness:mask:cell_region
form_factor:mask:cell_region
solidity:mask:cell_region
hole_ratio:mask:cell_region
area:mask:dna_region
filled_area:mask:dna_region
perimeter:mask:dna_region
major_axis_length:mask:dna_region
minor_axis_length:mask:dna_region
aspect_ratio:mask:dna_region
eccentricity:mask:dna_region
orientation:mask:dna_region
compactness:mask:dna_region
roundness:mask:dna_region
form_factor:mask:dna_region
solidity:mask:dna_region
hole_ratio:mask:dna_region
area:mask:nondna_region
filled_area:mask:nondna_region
perimeter:mask:nondna_region
major_axis_length:mask:nondna_region
minor_axis_length:mask:nondna_region
aspect_ratio:mask:nondna_region
eccentricity:mask:nondna_region
orientation:mask:nondna_region
compactness:mask:nondna_region
roundness:mask:nondna_region
form_factor:mask:nondna_region
solidity:mask:nondna_region
hole_ratio:mask:nondna_region
area:mask:DNA_object
filled_area:mask:DNA_object
perimeter:mask:DNA_object
major_axis_length:mask:DNA_object
minor_axis_length:mask:DNA_object
aspect_ratio:mask:DNA_object
eccentricity:mask:DNA_object
orientation:mask:DNA_object
compactness:mask:DNA_object
roundness:mask:DNA_object
form_factor:mask:DNA_object
solidity:mask:DNA_object
hole_ratio:mask:DNA_object
area:mask:Actin_object
filled_area:mask:Actin_object
perimeter:mask:Actin_object
major_axis_length:mask:Actin_object
minor_axis_length:mask:Actin_object
aspect_ratio:mask:Actin_object
eccentricity:mask:Actin_object
orientation:mask:Actin_object
compactness:mask:Actin_object
roundness:mask:Actin_object
form_factor:mask:Actin_object
solidity:mask:Actin_object
hole_ratio:mask:Actin_object
area:mask:Tubulin_object
filled_area:mask:Tubulin_object
perimeter:mask:Tubulin_object
major_axis_length:mask:Tubulin_object
minor_axis_length:mask:Tubulin_object
aspect_ratio:mask:Tubulin_object
eccentricity:mask:Tubulin_object
orientation:mask:Tubulin_object
compactness:mask:Tubulin_object
roundness:mask:Tubulin_object
form_factor:mask:Tubulin_object
solidity:mask:Tubulin_object
hole_ratio:mask:Tubulin_object
obj_number:mask:DNA_object
obj_mean_total_area:mask:DNA_object
obj_stddev_total_area:mask:DNA_object
obj_number:mask:Actin_object
obj_mean_total_area:mask:Actin_object
obj_stddev_total_area:mask:Actin_object
obj_number:mask:Tubulin_object
obj_mean_total_area:mask:Tubulin_object
obj_stddev_total_area:mask:Tubulin_object
fraction_total_intensity:DNA:dna_region-cell_region
total_intensity:DNA:dna_region
mean_intensity:DNA:dna_region
stddev_intensity:DNA:dna_region
cv_intensity:DNA:dna_region
skewness_intensity:DNA:dna_region
kurtosis_intensity:DNA:dna_region
fraction_total_intensity:DNA:DNA_object-cell_region
total_intensity:DNA:DNA_object
mean_intensity:DNA:DNA_object
stddev_intensity:DNA:DNA_object
cv_intensity:DNA:DNA_object
skewness_intensity:DNA:DNA_object
kurtosis_intensity:DNA:DNA_object
fraction_total_intensity:DNA:Actin_object-cell_region
total_intensity:DNA:Actin_object
mean_intensity:DNA:Actin_object
stddev_intensity:DNA:Actin_object
cv_intensity:DNA:Actin_object
skewness_intensity:DNA:Actin_object
kurtosis_intensity:DNA:Actin_object
fraction_total_intensity:DNA:Tubulin_object-cell_region
total_intensity:DNA:Tubulin_object
mean_intensity:DNA:Tubulin_object
stddev_intensity:DNA:Tubulin_object
cv_intensity:DNA:Tubulin_object
skewness_intensity:DNA:Tubulin_object
kurtosis_intensity:DNA:Tubulin_object
fraction_obj_intensity:DNA:dna_region-DNA_object
fraction_obj_intensity:DNA:dna_region-Actin_object
fraction_obj_intensity:DNA:dna_region-Tubulin_object
total_intensity:Actin:cell_region
mean_intensity:Actin:cell_region
stddev_intensity:Actin:cell_region
cv_intensity:Actin:cell_region
skewness_intensity:Actin:cell_region
kurtosis_intensity:Actin:cell_region
fraction_total_intensity:Actin:dna_region-cell_region
total_intensity:Actin:dna_region
mean_intensity:Actin:dna_region
stddev_intensity:Actin:dna_region
cv_intensity:Actin:dna_region
skewness_intensity:Actin:dna_region
kurtosis_intensity:Actin:dna_region
fraction_total_intensity:Actin:nondna_region-cell_region
total_intensity:Actin:nondna_region
mean_intensity:Actin:nondna_region
stddev_intensity:Actin:nondna_region
cv_intensity:Actin:nondna_region
skewness_intensity:Actin:nondna_region
kurtosis_intensity:Actin:nondna_region
fraction_total_intensity:Actin:DNA_object-cell_region
total_intensity:Actin:DNA_object
mean_intensity:Actin:DNA_object
stddev_intensity:Actin:DNA_object
cv_intensity:Actin:DNA_object
skewness_intensity:Actin:DNA_object
kurtosis_intensity:Actin:DNA_object
fraction_total_intensity:Actin:Actin_object-cell_region

Supplementary Table S2 : Feature list for the HeLa siRNA dataset (continue)

total_intensity:Actin:Actin_object
mean_intensity:Actin:Actin_object
stddev_intensity:Actin:Actin_object
cv_intensity:Actin:Actin_object
skewness_intensity:Actin:Actin_object
kurtosis_intensity:Actin:Actin_object
fraction_total_intensity:Actin:Tubulin_object-cell_region
total_intensity:Actin:Tubulin_object
mean_intensity:Actin:Tubulin_object
stddev_intensity:Actin:Tubulin_object
cv_intensity:Actin:Tubulin_object
skewness_intensity:Actin:Tubulin_object
kurtosis_intensity:Actin:Tubulin_object
fraction_obj_intensity:Actin:dna_region-DNA_object
fraction_obj_intensity:Actin:nondna_region-DNA_object
fraction_obj_intensity:Actin:dna_region-Actin_object
fraction_obj_intensity:Actin:nondna_region-Actin_object
fraction_obj_intensity:Actin:dna_region-Tubulin_object
fraction_obj_intensity:Actin:nondna_region-Tubulin_object
total_intensity:Tubulin:cell_region
mean_intensity:Tubulin:cell_region
stddev_intensity:Tubulin:cell_region
cv_intensity:Tubulin:cell_region
skewness_intensity:Tubulin:cell_region
kurtosis_intensity:Tubulin:cell_region
fraction_total_intensity:Tubulin:dna_region-cell_region
total_intensity:Tubulin:dna_region
mean_intensity:Tubulin:dna_region
stddev_intensity:Tubulin:dna_region
cv_intensity:Tubulin:dna_region
skewness_intensity:Tubulin:dna_region
kurtosis_intensity:Tubulin:dna_region
fraction_total_intensity:Tubulin:nondna_region-cell_region
total_intensity:Tubulin:nondna_region
mean_intensity:Tubulin:nondna_region
stddev_intensity:Tubulin:nondna_region
cv_intensity:Tubulin:nondna_region
skewness_intensity:Tubulin:nondna_region
kurtosis_intensity:Tubulin:nondna_region
fraction_total_intensity:Tubulin:DNA_object-cell_region
total_intensity:Tubulin:DNA_object
mean_intensity:Tubulin:DNA_object
stddev_intensity:Tubulin:DNA_object
cv_intensity:Tubulin:DNA_object
skewness_intensity:Tubulin:DNA_object
kurtosis_intensity:Tubulin:DNA_object
fraction_total_intensity:Tubulin:Actin_object-cell_region
total_intensity:Tubulin:Actin_object
mean_intensity:Tubulin:Actin_object
stddev_intensity:Tubulin:Actin_object
cv_intensity:Tubulin:Actin_object
skewness_intensity:Tubulin:Actin_object
kurtosis_intensity:Tubulin:Actin_object
fraction_total_intensity:Tubulin:Tubulin_object-cell_region
total_intensity:Tubulin:Tubulin_object
mean_intensity:Tubulin:Tubulin_object
stddev_intensity:Tubulin:Tubulin_object
cv_intensity:Tubulin:Tubulin_object
skewness_intensity:Tubulin:Tubulin_object
kurtosis_intensity:Tubulin:Tubulin_object
fraction_obj_intensity:Tubulin:dna_region-DNA_object
fraction_obj_intensity:Tubulin:nondna_region-DNA_object
fraction_obj_intensity:Tubulin:dna_region-Actin_object
fraction_obj_intensity:Tubulin:nondna_region-Actin_object
fraction_obj_intensity:Tubulin:dna_region-Tubulin_object
fraction_obj_intensity:Tubulin:nondna_region-Tubulin_object
total_intensity_ratio:DNA-Actin:dna_region-cell_region
total_intensity_ratio:DNA-Actin:dna_region-dna_region
total_intensity_ratio:DNA-Actin:dna_region-nondna_region
total_intensity_ratio:DNA-Tubulin:dna_region-cell_region
total_intensity_ratio:DNA-Tubulin:dna_region-dna_region
total_intensity_ratio:DNA-Tubulin:dna_region-nondna_region
total_intensity_ratio:Actin-DNA:cell_region-dna_region
total_intensity_ratio:Actin-Actin:cell_region-dna_region
total_intensity_ratio:Actin-Actin:cell_region-nondna_region
total_intensity_ratio:Actin-Tubulin:cell_region-cell_region
total_intensity_ratio:Actin-Tubulin:cell_region-dna_region
total_intensity_ratio:Actin-Tubulin:cell_region-nondna_region
total_intensity_ratio:Actin-DNA:dna_region-dna_region
total_intensity_ratio:Actin-Actin:dna_region-cell_region
total_intensity_ratio:Actin-Actin:dna_region-nondna_region
total_intensity_ratio:Actin-Tubulin:dna_region-cell_region
total_intensity_ratio:Actin-Tubulin:dna_region-dna_region
total_intensity_ratio:Actin-Tubulin:dna_region-nondna_region
total_intensity_ratio:Actin-DNA:nondna_region-dna_region
total_intensity_ratio:Actin-Actin:nondna_region-cell_region
total_intensity_ratio:Actin-Actin:nondna_region-dna_region
total_intensity_ratio:Actin-Tubulin:nondna_region-cell_region
total_intensity_ratio:Actin-Tubulin:nondna_region-dna_region
total_intensity_ratio:Actin-Tubulin:nondna_region-nondna_region
total_intensity_ratio:Tubulin-DNA:cell_region-dna_region
total_intensity_ratio:Tubulin-Actin:cell_region-cell_region
total_intensity_ratio:Tubulin-Actin:cell_region-dna_region
total_intensity_ratio:Tubulin-Actin:cell_region-nondna_region
total_intensity_ratio:Tubulin-Tubulin:cell_region-dna_region
total_intensity_ratio:Tubulin-Tubulin:cell_region-nondna_region
total_intensity_ratio:Tubulin-DNA:dna_region-dna_region
total_intensity_ratio:Tubulin-Actin:dna_region-cell_region
total_intensity_ratio:Tubulin-Actin:dna_region-dna_region
total_intensity_ratio:Tubulin-Actin:dna_region-nondna_region
total_intensity_ratio:Tubulin-Tubulin:dna_region-cell_region
total_intensity_ratio:Tubulin-Tubulin:dna_region-nondna_region
total_intensity_ratio:Tubulin-DNA:nondna_region-dna_region
total_intensity_ratio:Tubulin-Actin:nondna_region-cell_region
total_intensity_ratio:Tubulin-Actin:nondna_region-dna_region
total_intensity_ratio:Tubulin-Actin:nondna_region-nondna_region
total_intensity_ratio:Tubulin-Tubulin:nondna_region-cell_region
total_intensity_ratio:Tubulin-Tubulin:nondna_region-dna_region
ccorr:DNA-Actin:cell_region
ccorr_normed:DNA-Actin:cell_region
ccoeff:DNA-Actin:cell_region
ccoeff_normed:DNA-Actin:cell_region
ccorr:DNA-Tubulin:cell_region
ccorr_normed:DNA-Tubulin:cell_region
ccoeff:DNA-Tubulin:cell_region
ccoeff_normed:DNA-Tubulin:cell_region
ccorr:Actin-Tubulin:cell_region
ccorr_normed:Actin-Tubulin:cell_region
ccoeff:Actin-Tubulin:cell_region
ccoeff_normed:Actin-Tubulin:cell_region
ccorr:DNA-Actin:dna_region
ccorr_normed:DNA-Actin:dna_region
ccoeff:DNA-Actin:dna_region
ccoeff_normed:DNA-Actin:dna_region
ccorr:DNA-Tubulin:dna_region
ccorr_normed:DNA-Tubulin:dna_region
ccoeff:DNA-Tubulin:dna_region
ccoeff_normed:DNA-Tubulin:dna_region
ccorr:Actin-Tubulin:dna_region
ccorr_normed:Actin-Tubulin:dna_region
ccoeff:Actin-Tubulin:dna_region
ccoeff_normed:Actin-Tubulin:dna_region
ccorr:DNA-Actin:nondna_region
ccorr_normed:DNA-Actin:nondna_region
ccoeff:DNA-Actin:nondna_region
ccoeff_normed:DNA-Actin:nondna_region
ccorr:DNA-Tubulin:nondna_region
ccorr_normed:DNA-Tubulin:nondna_region
ccoeff:DNA-Tubulin:nondna_region
ccoeff_normed:DNA-Tubulin:nondna_region
ccorr:Actin-Tubulin:nondna_region
ccorr_normed:Actin-Tubulin:nondna_region
ccoeff:Actin-Tubulin:nondna_region
ccoeff_normed:Actin-Tubulin:nondna_region
