# Supplementary material for: cellXpress: a fast and user-friendly software platform for profiling cellular phenotypes
Source: BMC Bioinformatics. 2013 Oct 22;14(Suppl 16):S4. doi: 10.1186/1471-2105-14-S16-S4 (PMC3853218; doi:10.1186/1471-2105-14-S16-S4)
Supplement: Additional file 3 — Total processing time for cell segmentation (unit = second, k = frame number, CP = CellProfiler, cX = cellXpress) [file 1471-2105-14-S16-S4-S3.PDF]

Supplementary Table S3: Total processing time for cell segmentation (unit=second, k=frame number, CP=CellProfiler, cX=*cellXpress*)

	**Kc167 (k=1)**			**HT29 (k=55)**			**HeLa (k=176)**		
**No.**	**CP**	**Fiji**	**cX**	**CP**	**Fiji**	**cX**	**CP**	**Fiji**	**cX**
**1**	8	3.78	1.71	97	60.89	5.38	511	221.49	30.49
**2**	7	3.61	1.56	99	59.65	5.52	514	219.72	28.67
**3**	8	3.67	1.62	98	59.59	6.02	509	220.34	37.47
**4**	8	3.75	1.73	98	58.75	5.31	516	223.32	33.95
**5**	7	3.83	1.65	97	59.27	5.73	514	218.38	29.77
**Mean**	**7.6**	**3.73**	**1.65**	**97.8**	**59.63**	**5.59**	**512.8**	**220.65**	**32.07**
